# Link-Based Similarity Measures Using Reachability Vectors

**DOI:** 10.1155/2014/741608

**Published:** 2014-02-18

**Authors:** Seok-Ho Yoon, Ji-Soo Kim, Jiwoon Ha, Sang-Wook Kim, Minsoo Ryu, Ho-Jin Choi

**Affiliations:** ^1^Department of Electronics and Computer Engineering, Hanyang University, Seoul 133-791, Republic of Korea; ^2^Department of Computer and Software, Hanyang University, Seoul 133-791, Republic of Korea; ^3^Department of Computer Science, KAIST, Daejeon 305-701, Republic of Korea

## Abstract

We present a novel approach for computing link-based similarities among objects accurately by utilizing the link information pertaining to the objects involved. We discuss the problems with previous link-based similarity measures and propose a novel approach for computing link based similarities that does not suffer from these problems. In the proposed approach each target object is represented by a vector. Each element of the vector corresponds to all the objects in the given data, and the value of each element denotes the weight for the corresponding object. As for this weight value, we propose to utilize the probability of reaching from the target object to the specific object, computed using the “Random Walk with Restart” strategy. Then, we define the similarity between two objects as the cosine similarity of the two vectors. In this paper, we provide examples to show that our approach does not suffer from the aforementioned problems. We also evaluate the performance of the proposed methods in comparison with existing link-based measures, qualitatively and quantitatively, with respect to two kinds of data sets, scientific papers and Web documents. Our experimental results indicate that the proposed methods significantly outperform the existing measures.

## 1. Introduction

Similarities among objects provide useful information to wide application areas such as ranking Web documents [1], detecting duplicate documents [2], comparing user profiles in e-commerce recommendation systems [3], searching for similar papers in literature databases [4, 5], and the like. Accurate computation of similarities among objects is crucial to the success of these applications [6, 7]. For example, the collaborative filtering technique used in an e-commerce system makes recommendations of goods or products to a user by choosing from the purchase list of those users deemed similar to that user. In order to search for the “similar” users, the system needs to compute the similarities among users [3]. If the similarities are not accurate, the user would get recommendations with unwanted items.

Existing similarity measures can be classified into either content-based or link-based ones [8]. Content-based measures compute similarities among objects by comparing the contents of the objects involved, such as texts and multimedia. In the various types of contents, these measures mainly utilize the textual information, which is easier to analyze than other types. Measures for computing the similarities among objects using textual information are referred to as text-based similarity measures [9, 10]. Cosine similarity, SVD, LDA, LSI-based similarity measures, and *χ*-Sim belong to this category [11]. In a text-based similarity measure, the similarity between two objects becomes higher in general when the two objects have more words in common.

On the other hand, link-based measures represent the relationships among objects as links and compute the similarities using the link information. The more neighbors two objects have in common, the higher the similarity between the two becomes. Typical link-based measures include Cocitation [12], Bibliographic coupling [13], Amsler [14], SimRank [15], rvs-SimRank [6], and P-Rank [6]. As compared to text-based measures, link-based measures have recently been paid attention to for the merits of language-independency, good performance, and being able to produce results appealing to human intuition [6, 16]. For these reasons, the work in this paper will also focus on the link-based similarity measures.

Among the existing link-based measures, SimRank is well known and has driven a large number of subsequent studies which proposed variations of SimRank [6, 17] or investigated performance speed-ups of it [18–25]. The basic principle of SimRank states that “two objects are similar if they are related to similar objects.” SimRank computes the similarity between two objects, say *x* and *y*, by recursively computing average of all the similarities between every object pointing to *x* and every object pointing to *y* [15]. In other words, SimRank can be better explained in terms of what we call the “pair-wise” and the “level-wise” computation models. The pair-wise model is to compute the similarity between *x* and *y* by averaging all the similarities computed between every neighbor of *x* and every neighbor of *y*, whereas the level-wise model is to compute the similarity by utilizing only those other objects located in the same distance from *x* and *y* level by level.

We believe that any similarity measure such as SimRank which uses both the pair-wise and level-wise models cannot accurately compute the similarity between two objects. For instance, the pair-wise model would still compute the similarities between each neighbor of *x* and each neighbor of *y* even if the entire set of neighbors of *x* and *y* is exactly the same. Consequently, the pair-wise model in general yields biased results in that the similarity between two objects having a large number of links tends to become lower than the similarity between two objects having a small number of links [18, 26]. On the other hand, the level-wise model focuses only on those neighbor objects linked directly to the two objects “at the same level,” that is, in the graph, only considering those objects located at the same distance from the two objects. Consequently, this model cannot consider all of the objects linked directly or indirectly to the two target objects.

This paper proposes a new link-based similarity measure that does not suffer from the aforementioned problems with the pair-wise and level-wise models. In the proposed measure, we represent each object, say *x*, as a vector. The elements of the vector (for *x*) correspond to all the objects (including *x* itself) in the given universe, and the value of each element denotes the weight (with respect to *x*) for the particular object corresponding to the element. To obtain this weight value, we propose to utilize the probability of reaching from the object *x* to the particular object, computed using the “Random Walk with Restart” strategy. Then, we define the similarity between two objects as the cosine similarity [9] between the two vectors representing the two objects. This approach resembles the text-based similarity measures using the cosine similarity for computing similarity between documents, where a document is represented by a vector; each element of the vector corresponds to each word in the universe of all documents; and the value of each element denotes the frequency of the corresponding word in the target document.

Our approach does not suffer from the problem with the pair-wise model because it computes the similarity between two vectors by multiplying only the values of the corresponding elements in the two vectors, not trying every possible element pair between the two vectors. Moreover, the approach can also consider all the objects linked directly or indirectly to the two target objects, reflecting the degree of “closeness” between objects in the form of reachability between objects. Thus, it does not suffer from the problem with the level-wise model, either. In this paper, we will develop two methods to implement our approach. The first method will generate the vectors representing objects using inlinks and outlinks separately then merges two vectors to compute the similarity. The second method will not distinguish inlinks from outlinks but convert them together into undirected links to generate the vectors. The effectiveness of both methods will be demonstrated by examples, showing that they do not suffer from problems of the pair-wise and level-wise models.

The paper also evaluates the performance of the proposed methods in comparison with existing link-based measures, qualitatively and quantitatively, using the data sets of scientific papers and Web documents, as two exemplary types of data having link information. Our experimental results indicate that the proposed methods generally outperform the existing measures for both types of data.

The rest of this paper is organized as follows.  Section 2 summarizes existing link-based similarity measures.  Section 3 explains our research motivations, and Section 4 describes the proposed approach and methods in detail.  Section 5 presents the experimental results to validate the performance of the proposed methods.  Section 6 concludes the paper.

## 2. Related Work

Existing link-based similarity measures include Cocitation, Bibliographic coupling, Amsler, SimRank, rvs-SimRank, and P-Rank. While Cocitation, Bibliographic coupling, and Amsler were originally devised to deal with scientific papers [6], they have also been applied to other types of data such as Web documents which have link information [27, 28]. On the other hand, SimRank, rvs-SimRank, and P-Rank were originally proposed to deal with objects of any kind having link information [6, 15].

Cocitation computes the similarity between two objects based on the number of objects which commonly point to the two. As a result, the similarity between two objects becomes higher as the number of “commonly pointing” objects gets larger [12]. This concept is described as follows, where *x* and *y* denote objects, *S*(*x*, *y*) the similarity between *x* and *y*, and *I*(*x*) and *I*(*y*) the sets of objects pointing to *x* and *y*, respectively:

(1) $$\begin{array}{c} S\left(x,y\right)=\left|I\left(x\right)\cap I\left(y\right)\right|. \end{array}$$

Bibliographic coupling computes the similarity between two objects based on the number of objects which are commonly pointed by the two [13]. This is described as follows, where *O*(*x*) and *O*(*y*) represent the sets of objects pointed by *x* and *y*, respectively:

(2) $$\begin{array}{c} S\left(x,y\right)=\left|O\left(x\right)\cap O\left(y\right)\right|. \end{array}$$

The above two measures, namely, Cocitation and Bibliographic coupling, are combined together by Amsler, which defines the similarity between two objects as a weighted sum of the two similarities computed by Cocitation and Bibliographic coupling as described in (3), where *λ* represents the factor to balance the weights between the two similarities involved. In general, *λ* is set to 0.5 to assign equal weights between the two [6, 14]:

(3) $$\begin{array}{c} S\left(x,y\right)=\lambda \times \left(\left|I\left(x\right)\cap I\left(y\right)\right|\right)+\left(1-\lambda \right)\times \left(\left|O\left(x\right)\cap O\left(y\right)\right|\right). \end{array}$$

**Figure 1:**
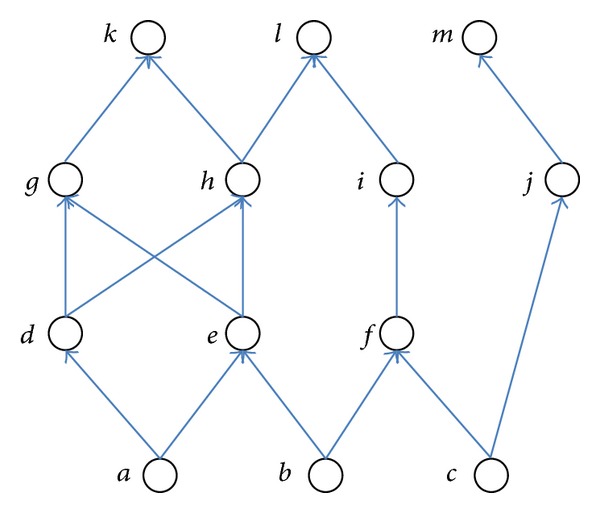
An example graph.

The concepts of these three measures can be illustrated using the graph in Figure 1, which represents objects as nodes and reference relationships among objects as links. The similarity between objects *g* and *h* in the graph will become 2, when computed by Cocitation, since there exist two objects (i.e., *d* and *e*) commonly pointing to *g* and *h*. On the other hand, the similarity will become 1, when computed by Bibliographic coupling, since there exists only one object (i.e., *k*) commonly pointed by *g* and *h*. Finally, Amsler would compute the similarity between *g* and *h* to be 1.5, assuming the same weights (*λ* = 0.5) among both measures of the Cocitation and Bibliographic coupling (i.e., 1.5 = 0.5 × 2 + 0.5 × 1 using (3)).

Note that the similarities such computed by Cocitation, Bibliographic coupling, and Amsler in general tend to become larger as the number of links among the objects becomes larger. This phenomenon can be normalized by dividing the similarities by the size of the union of the sets of objects commonly pointing to (or pointed by) the two objects, yielding the resulting similarity to be between 0 and 1 [29].

SimRank uses the concept that “two objects are similar if they are related to similar objects.” In the graph of Figure 1, for example, the similarity between objects *h* and *i* would be computed as zero, that is, interpreted as “not similar at all,” using Cocitation since no object exists which commonly points to both *h* and *i*. Nevertheless, these two objects could be seen as similar to some degree in that *h* and *i* are separately pointed by two “similar” objects, *e* and *f*, respectively, because *e* and *f* are commonly pointed by *b*. SimRank exploits such a concept by recursively computing the similarity between two objects, say *x* and *y*, as the average of all the similarities between every object pointing to *x* and every object pointing to *y*. This concept of SimRank is described by (4), where *x* and *y* denote objects, *S*(*x*, *y*) the similarity between *x* and *y*, and *I*(*x*) and *I*(*y*) the sets of objects pointing to *x* and *y*, respectively. *I*_*i*_(*x*) is the *i*th object in the list of objects pointing to *x*, and *C* is the decay factor having the value between 0 and 1. The decay factor reduces the weights of the computed similarity as the iterations get deeper. As shown by the following, SimRank yields the similarity as a value between 0 and 1, by normalizing the summation of the similarities of all pairs of objects in the Cartesian product of the two sets, *I*(*x*) and *I*(*y*), by its cardinality [15]:

(4) $$\begin{array}{c} S\left(x,y\right)=\frac{C}{\left|I\left(x\right)\right|\left|I\left(y\right)\right|}\overset{\left|I(x)\right|}{\underset{i=1}{\sum }}\overset{\left|I(y)\right|}{\underset{j=1}{\sum }}S\left(I_{i}\left(x\right),I_{j}\left(y\right)\right). \end{array}$$

In a sense, SimRank expands the Cocitation to a broader scope of neighbor objects in the similarity definition, so as to count not just the adjacent objects directly linked to the two target objects (as in Cocitation) but also to consider the effects of all other objects indirectly linked (through the recursive computation). In a similar manner, [6] expands Bibliographic coupling to yield rvs-SimRank, and Amsler to yield P-Rank. The rvs-SimRank is expressed by (5), which differs from (4) of SimRank only in that it uses outlinks instead of inlinks. The P-Rank is expressed by (6), which computes the similarity as a weighted sum of the two similarities obtained by SimRank and rvs-SimRank, respectively, in each iteration step. Consider the following:

(5) $$\begin{array}{c} S\left(x,y\right)=\frac{C}{\left|O\left(x\right)\right|\left|O\left(y\right)\right|}\overset{\left|O(x)\right|}{\underset{i=1}{\sum }}\;\overset{\left|O(y)\right|}{\underset{j=1}{\sum }}S\left(O_{i}\left(x\right),O_{j}\left(y\right)\right), \end{array}$$

(6) S(x,y) =λ×C|I(x)||I(y)|∑i=1|I(x)| ∑j=1|I(y)|S(Ii(x),Ij(y))  +(1−λ)×C|O(x)||O(y)|∑i=1|O(x)| ∑j=1|O(y)|S(Oi(x),Oj(y)).

In another direction, various approaches have sought improving the accuracy of existing measures [4, 18, 26]. Reference [18] proposes to apply Jaccard coefficient to the SimRank in order to remedy the phenomenon that the similarities tend to become lower among the objects having a larger number of links. Reference [26] proposes to improve the accuracy of SimRank by taking the average of the similarities only between the maximally matching neighbor objects across the two groups associated with the two target objects, in order to resolve the problem indicated in [18].

Many of these approaches have also been investigated to improve the speed of existing measures [18, 20, 21]. Reference [18] suggests to improve the performance of SimRank by proposing to construct first a fingerprint tree for each object and then use such trees to approximate the similarity to be obtained by SimRank. Reference [21] proposes to reduce the time and space complexity of SimRank by utilizing a tree structure called SimTree, which allows storing directly the similarities among similar objects but computing the similarities among dissimilar objects using the path information of the tree. Reference [20] aims to compute the similarity between two objects using SimRank in online real-time by suggesting to consider only those objects related directly to the two target objects rather than computing similarities involved in all objects. Reference [24] investigates a method to run SimRank in parallel using GPGPU (general-purpose computation on graphics processors) and a method to approximately compute the similarity in a dynamic graph using uncoupling Markov chains.

## 3. Motivation

In this section, we discuss the problems with existing link-based similarity measures. First, we cast existing measures into a method combining two computation models which we will call “pair-wise” and “level-wise,” and explain the inherent difficulties in these models. We then analyze and illustrate the limitations of three representative existing methods, namely, rvs-SimRank, SimRank, and P-Rank, showing that each of these methods actually combines the pair-wise and level-wise models.

### 3.1. Pair-Wise and Level-Wise Computation Models

In the graph of Figure 1 again, SimRank would compute the similarity between *k* and *l* by taking the average of the four similarities obtained from the four pairs (*g*, *h*), (*g*, *i*), (*h*, *h*), and (*h*, *i*), namely, the Cartesian product of the set {*g*, *h*}, the objects directly “in-linked” to *k*, with the set {*h*, *i*}, and the objects directly “in-linked” to *l*. We call such a way of pairing and averaging out the similarities for all such pairs the “pair-wise” computational model. In this example, the similarity between *k* and *l*, *S*(*k*, *l*), becomes the average of *S*(*g*, *h*), *S*(*g*, *i*), *S*(*h*, *h*), and *S*(*h*, *i*). Here, *S*(*h*, *h*) = 1 by definition, and *S*(*g*, *h*), *S*(*g*, *i*), and *S*(*h*, *i*) should also be computed in turn by SimRank in a recursive manner using the same pair-wise model. That is, *s*(*g*, *h*) is computed from the Cartesian product of *g*'s neighbors {*d*, *e*} and *h*'s neighbors {*d*, *e*}, *S*(*g*, *i*) from *g*'s neighbors {*d*, *e*} and *i*'s neighbors {*f*}, and *S*(*h*, *i*) from *h*'s neighbors {*d*, *e*} and *i*'s neighbors {*f*}, using the pair-wise model. In this way, the recursive process continues and gets deeper, using all the objects linked directly or indirectly to the two target objects for which the similarity is being computed.

On the other hand, such a model can be seen as computing the similarity between two objects by utilizing only those other objects located in the same distance from the two targets level by level. In Figure 1, for example, the model computes the similarity between *k* and *l* by utilizing the objects with distance 1 (namely, nodes *g*, *h*, and *i*) in the first round, then the objects with distance 2 (namely, *d*, *e*, and *f*) in the second round, and then the objects with distance 3 (namely, *a*, *b*, and *c*) in the third round. We call such process the “level-wise” computation model.

### 3.2. Problem of Pair-Wise Computation Model

**Figure 2:**
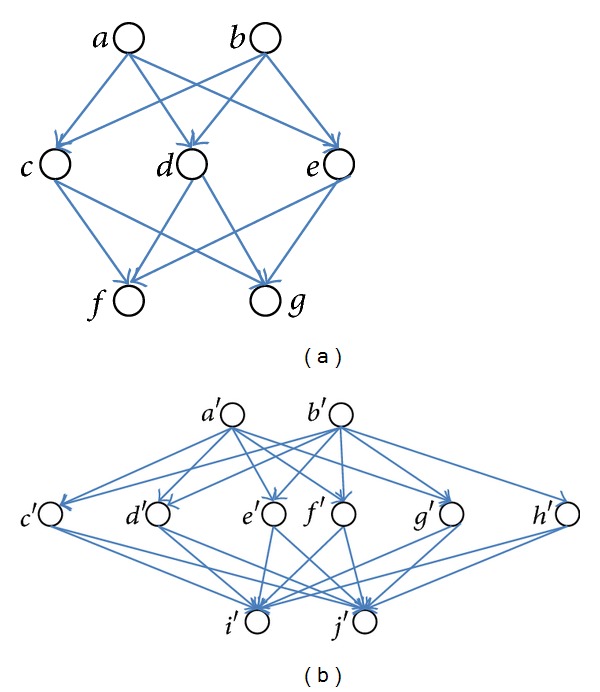
Example graphs to illustrate the problem with the pair-wise model.

Figure 2 illustrates the problem of the pair-wise model which induces the phenomenon that the similarities among objects having more links tend to become lower than those among objects having less links. In the graphs of Figure 2 again, nodes represent objects and links represent the reference relationships among objects. For example, objects *a* and *b* in Figure 2(a) both refer to the same three objects, *c*, *d*, and *e*, all of which in turn refer to the same two objects, *f* and *g*. Similarly, objects *a*′ and *b*′ in Figure 2(b) both refer to the same objects, all of which in turn refer to the same objects. The only difference between these two graphs is that the numbers of links from objects *a*′ and *b*′ are larger (i.e., six links each) than those from objects *a* and *b* (i.e., three links each). Intuitively, the similarity between *a* and *b* should be the same as that between *a*′ and *b*′, by observing that *a* and *b* both refer to the same objects, and also *a*′ and *b*′ both refer to the same objects. Existing methods, however, produce different results.

Table 1 shows the resulting similarities between *a* and *b* and between *a*′ and *b*′ as to be computed by three existing methods, rvs-SimRank, SimRank, and P-Rank, assuming *C* and *λ* to be 0.7 and 0.5 in formulas (4), (5), and (6). Two entries of the table indicate that the computed similarities between *a* and *b* are higher than those between *a*′ and *b*′, which is counterintuitive. Considering that the difference in the numbers of links between the two graphs in this example is only as small as four, one can imagine that this phenomenon would become clearer as the numbers of links get larger, for example, with such data set as scientific papers or Web documents. In the papers data sets, for example, well known papers tend to have many reference links because they will in general get substantially larger numbers of citations than ordinary papers, and similarly in the Web documents data set, portal sites tend to have many links because they are referenced more frequently than ordinary sites. We envisage that, for these domains, those methods which use the pair-wise computation model cannot compute the similarity between objects accurately.

### 3.3. Problem of Level-Wise Computation Model

**Figure 3:**
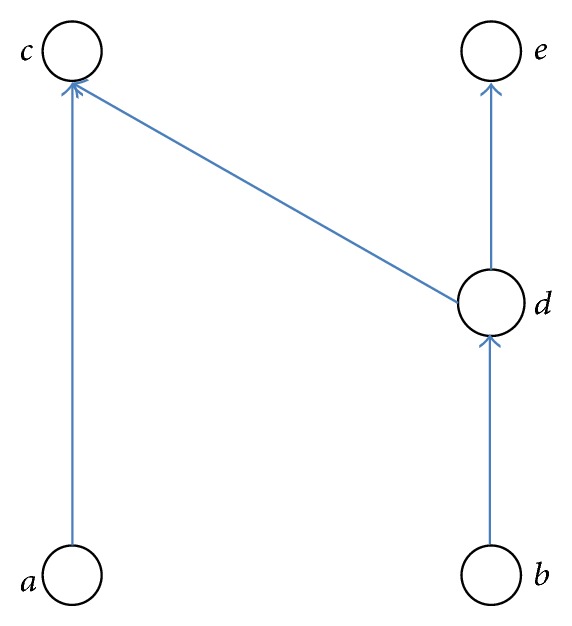
An example graph to illustrate the problem with the level-wise model.

The level-wise computation model computes the similarity between two objects by utilizing only those objects in the graph in the same distance from the two. The problem with this model can be illustrated using Figure 3, where again nodes represent objects and directed links represent the reference relationships between objects. Intuitively, we can say that two objects *a* and *b* are similar to some degree as they share, *though indirectly*, an object, *c*, in common. All of the aforementioned three methods, however, compute the similarity between these two objects, *a* and *b*, to be 0. For this case, one would expect to compute the similarity by considering all objects directly and indirectly linked to *a* (namely, *c*) and all objects directly and indirectly linked to *b* (namely, *c*, *d*, and *e*). In fact, however, any methods which utilize the level-wise model will compute *s*(*a*, *b*) by only using *c* (as *a*'s neighbor) and *d* (as *b*'s neighbor). Here, *s*(*c*, *d*) should also be computed by taking the average of the similarities for all possible pairs across *c*'s neighbor objects and *d*'s neighbor objects. In this case, however, *c*'s neighbor does not exist, yielding *s*(*c*, *d*) to be 0 even though there still remain *d*'s neighbors, *c* and *e*, which should also be counted by some means. After all, *s*(*a*, *b*) will also become 0, indicating that they are not similar at all. From this example, we conclude that the level-wise model cannot compare the objects not located at the same distance and consequently cannot compute the similarity properly.

## 4. Proposed Methods

### 4.1. Main Concepts

In this section, we propose a novel link-based similarity measure. Our approach differs from the existing link-based methods in that we will not define the measure by combining the pair-wise and level-wise models, but use the concept of the cosine similarity. As expressed by (7), the cosine similarity is a measure for computing a similarity between a pair of vectors. Thus, in computing a similarity between objects using the cosine similarity, the features of an object are represented by the elements of a vector, and the weight to each feature is captured by the value of each element. For example, in case of computing a similarity between documents using cosine similarity, each document is represented by a vector, the words in the given universe of documents are denoted by the elements of the vector, and the frequency of each word in the document is indicated by the value of the corresponding element. Then, the similarity between two documents is computed by the similarity between the two corresponding vectors [9]:

(7) $$\begin{array}{c} s\left(x,y\right)=\frac{x\cdot y}{||x||||y||}=\frac{\sum_{i=1}^{n}\left(x_{i}\times y_{i}\right)}{\sqrt{\sum_{i=1}^{n}{\left(x_{i}\right)}^{2}}\times \sqrt{\sum_{i=1}^{n}{\left(y_{i}\right)}^{2}}}. \end{array}$$

In our approach, each object is represented as a vector, all other objects in the given universe as the elements of the vector, and some “weight value” to each object as the value of the corresponding element in the vector. As for the cosine similarity used by text-based measures discussed above, where words of higher-frequency get larger weight values by being treated as better characterizing features for a document, we need to define a measure to quantify the degree with which to determine how well the target object is characterized by each object in the universe. For this measure, we propose to use the degree of how close the two objects are located in the topological point of view.

Sun et al. [30] proposed a method for computing the probability of reaching from an object to another as the relevance between two objects, using the “Random Walk with Restart (RWR)” strategy. We adopt this strategy and use the reachability to an object from the target object as the weight value for its corresponding element of the vector representing the target object. Reachability becomes higher as the distance between two objects becomes shorter, and also when more paths exist between the two. Computing reachability using RWR is expressed by the following, where *P*_*A*_ represents an adjacency matrix column-normalizing the connectedness among objects, *u*_*a*_ a vector having reachability to each node starting from *a*, *q*_*a*_ a restart vector having the value 1 only for the starting node *a* and 0 for the rest, and *c* the restart probability:

(8) $$\begin{array}{c} \vec{u}=\left(1-c\right)P_{A}\vec{u_{a}}+c\vec{q_{a}}. \end{array}$$

Our approach does not suffer from the problem with the pair-wise model because it computes the similarity between two vectors by multiplying only the values of the corresponding elements in the two vectors, not trying every possible element pair between the two vectors. Moreover, the approach also generates the vectors by considering all the objects linked directly or indirectly to the two target objects. This mechanism is different from the pair-wise and level-wise models and does not suffer from the problems discussed in the previous section.

In this paper, we develop two methods to implement our approach. The two methods slightly differ only in their ways to compute the weights in the vector. One method computes the weights by first computing two values of reachability, one using only “inlinks” and the other using only “outlinks” separately, and then combining them together as a weighted sum. This method provides flexibility to a problem domain by allowing different weights to reachability using inlinks versus outlinks. The method, however, cannot compute reachability properly for those objects located closely with the target object, because links in both directions are not used together. In Figure 1, for example, nodes *a* and *b* are located closely but the reachability would become 0 when computed using only outlinks (or using only inlinks) from *a* or *b*. Another problem would be the difficulty in determining the appropriate weights for a vector generated purely using the inlinks, or purely using the outlinks. Thus, we develop the second method which computes reachability by ignoring the directions of inlinks and outlinks and converting them to undirected links before computing reachability. This method is advantageous in that it can compute appropriate reachability for every object. In the rest of this paper, we will call these two methods the “weightedSum” method and the “undirected” method, respectively. In the weightedSum method, in particular, the proper balance between the weights for inlinks and outlinks needs to be found domain by domain through experimentation.

### 4.2. Procedure of the Proposed Methods

**Figure 4:**
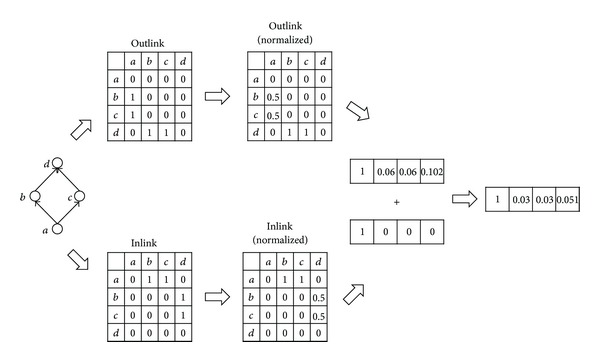
The procedure of generating a vector using the weightedSum method.

**Figure 5:**
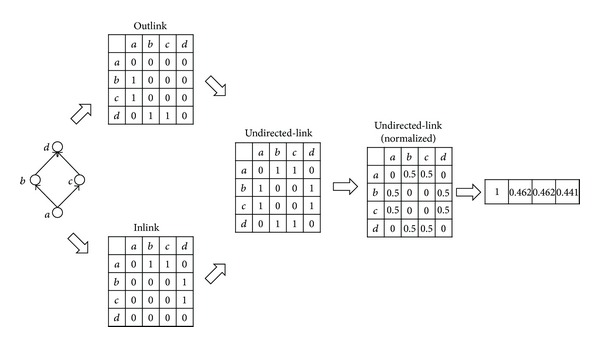
The procedure of generating a vector using the undirected method.

Basically, both of the proposed methods (1) construct vectors by computing reachability from the target object to all other objects and (2) compute the similarity between vectors by using the cosine similarity. The two methods differ only in the process of generating vectors, as illustrated by Figure 4 (for the weightedSum method) and Figure 5 (for the undirected method). The weightedSum method generates vectors in the following manner. First, two adjacency matrices are built with inlinks and outlinks and then column-normalized such that the sum of all values in a column becomes 1. Second, a vector is generated from the normalized matrix, and the weights are assigned to the elements in the vector by computing the reachability from the target object to every object using the RWR strategy. Similarly, the undirected method generates a vector by constructing a normalized adjacency matrix, ignoring the link directions this time, and assigning the reachability values to the elements of the vector in the same manner.

### 4.3. Complexity Analysis

The complexity of the proposed methods for computing similarities for *all pairs of given objects* can be analyzed as follows. First, time complexity of generation process of a vector for an object using the RWR strategy is *O*(*ke*) [31], where *e* represents the number of links and *k* the number of iterations for the matrix calculation to obtain converged values of reachability. Thus, time complexity of generation process for all objects *n* is *O*(*kne*), where *n* represents the number of objects. It is generally known that the constant number of such iterations would be sufficient to obtain converged values of reachability [6, 15]. Consequently, time complexity is reduced to *O*(*ne*). On the other hand, time complexity of the similarity calculation process is *O*(*n* ^3^). Combining them together, overall time complexity of both methods becomes *O*(*n* ^3^) + *O*(*ne*) = *O*(*n* ^3^). In practice, the weightedSum method will require double time than the undirected method because the former computes reachability separately for each direction of the links.

As for space complexity, *O*(*e*) space is required for storing the matrix to represent the relationships between objects, *O*(*n* ^2^) for storing vectors, and *O*(*n* ^2^) for storing the similarity measures between objects. Combining them together, overall space complexity becomes *O*(*n* ^2^). Again in practice, the weightedSum method will require double space than the other.

In comparison, time complexity of the three existing methods, namely, rvs-SimRank, SimRank, and P-Rank, is known to be *O*(*n* ^4^) and space complexity *O*(*n* ^2^) [6]. We believe the existing methods require more computation than our proposed methods because they adopt the pair-wise computation model in principle.

Moreover, the existing methods cannot compute the similarity between two given objects independently because they need to know the similarities among all objects (whether connected directly or indirectly to the target object) in order to compute the similarity between two target objects [20]. However, our approach does not require the similarities among all objects and hence can compute the similarity between any pair of objects independently, making it possible to parallelize the algorithm. Since our approach need not refer to the similarities among other objects, vectors for individual objects can be generated independently, and the similarity between any pair of objects can be computed separately, that is, in parallel. Time complexity of this parallelized version would become *O*(*n* ^3^/*m*) if *m* processors are utilized in parallel.

When we need to compute the similarity between only a particular pair of objects on-line, rather than computing the similarities among all objects [20], our methods can be made even more efficient by performing the computation of reachability on-line, through the inverse matrix as suggested by (9). That is, the vector for a given object can be generated by multiplying *q* to the inverse matrix of *Q* [30, 31]. Time complexity of this off-line computation of inverse matrix is *O*(*n* ^2.376^) [32], and the complexity of the on-line computation is *O*(*n*). Such an off-line approach tends to require relatively longer time and more space to handle the inverse matrix. Reference [31] suggests an approximation approach using low-rank approximation in order to keep balance between the off-line and on-line computation. This approach has advantages in both time and space complexity, suggesting improvement for on-line execution of our proposed approach. In conclusion, time complexity of our methods is lower than that of existing methods and space complexity is equal to that of existing methods in any case:

(9) u→=(1−c)PAua→+cqa→=(I−(1−c)PA)−1qa→=CQ−1qa→.

### 4.4. Discussions

The proposed methods do not suffer from the problems of the pair-wise and level-wise models discussed in Section 3. Let us compute the similarities between *a* and *b* and *a*′ and *b*′ in Figure 2 using our approach and check whether the results appeal to our intuition. We assume *λ* and *C* to be 0.5 and 0.7, respectively.  Table 2 shows the similarity results computed by the two proposed methods, together with those obtained by the three existing methods (shown already in Table 1) for comparison. Both of our methods have produced coinciding results that the similarity between *a* and *b*, and *a*′ and *b*′ is 1, saying that the two objects are regarded as the same. These results appeal to our intuition. In comparison, the results obtained by the existing methods indicate that the two objects are not the same. Since our approach does not use the pair-wise model but perform the computations among identical features, the proposed methods will produce results more appealing to our intuition than existing methods.

The proposed methods do not suffer from the problem of the level-wise model either. Let us compute the similarity between *a* and *b* in Figure 3 using our approach and check whether the results appeal to our intuition. Assuming *λ* and *C* to be 0.5 and 0.7, respectively, our approach has computed the similarity between *a* and *b* to be 0.28 using the weightedSum method, and 0.6 using the undirected method. For comparison, the existing methods would compute the similarity to be 0, as discussed in Section 3. We cannot tell if the results obtained by our approach are appropriate or not because the similarity is by nature a subjective value. Still, we can at least argue that our approach produces results which are more appealing to our intuition than the existing methods, that is, the similarity between *a* and *b* must not be 0 because they are related, indirectly, by some common objects. This difference has come from our strategy of considering for all the objects indirectly connected to the target objects at once, rather than using the level-wise model.

## 5. Experiments

In this section, we verify the effectiveness of our approach through experimentation. We will show and analyze quantitatively the experimental results obtained from applying the two proposed methods to practical application domains.

### 5.1. Experimentation Setup

We carried out experiments to verify the performance of the proposed methods with respect to scientific papers and Web documents, the two types of exemplary data sets having link information. For the experiments with the scientific papers, we used the data set consisting of the papers downloaded from DBLP (http://www.informatik.uni-trier.de/~ley/db/) and reference information among the papers crawled from Libra (http://academic.research.microsoft.com). In total, 44,800 papers and 126,281 references were used. For the experiments with the Web documents, we used the data set consisting of 1,227,038 Web pages with 11,164,829 hyperlinks in total, taken from the TREC (http://trec.nist.gov/data/t11.web.html) 2002 data. The experiments were carried out in a platform with Quad Core 2.67 GHz CPU, Windows 2008 Sever OS.

In the experiments, we aimed to evaluate the performance of the two proposed methods (weightedSum, undirected) in comparison with the three existing methods (rvs-SimRank, SimRank, and P-Rank) with the input values of 0.8, 0.5, and 0.15 for *C*, *λ*, and restart probability, respectively, as used frequently in the existing methods [6, 15, 30].

The method of experiments proceeded as follows. For the weightedSum method, we first assigned appropriate weights to the vectors generated with inlinks and outlinks by varying the weights and finding the ones that achieve the highest accuracy in the similarity through weightedSum method. In the experiments, we tried the weight values of 0, 0.1, 0.3, 0.5, 0.7, 0.9, and 1 for inlinks (note, the sum of in-link and out-link weights equals 1). Second, we evaluated the accuracy of each method qualitatively by examining 10 objects that the method computes as the most similar to a target object chosen arbitrarily. Finally, we measure the accuracy of each method quantitatively by comparing the obtained results with the true answers for each data set.

The measurement of the accuracy proceeded as follows. First, we chose one object in turn as the target object from the answer set. Then, we computed the recall [29] by extracting *m* objects (where *m* can be 10, 20, 30, 40, and 50) most similar to the target object according to each method. This process was repeated until every object in the set has been chosen as the target object. The average of all recall values obtained as such will be taken as the final accuracy value.

The answers of each data set were constructed in the following manner. For the experiments with scientific papers, we selected five well known areas (i.e., clustering, sequential pattern mining, spatial databases, link mining, and graph pattern mining) in a data mining text book [29] and obtained papers referenced in each section of these areas. We supposed that the reference papers in the same section are similar to one another. Thus, those papers in a section formed an answer set. Each answer set had 3 to 14 papers, and the total number of papers in all such answer sets was 106. For the experiments with Web documents, we used TREC 2002. TREC 2002 provides Web document sets related to specific keywords. We chose 9 Web document sets randomly from TREC 2002 and used the sets as the answer sets.

### 5.2. Domain of Scientific Papers

**Figure 6:**
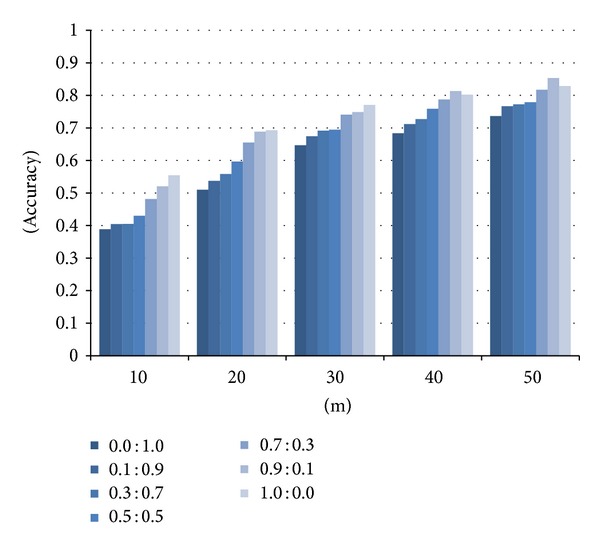
Accuracy of the weightedSum method with varying weights.

Figure 6 depicts the results showing the accuracy on the similarities among scientific papers obtained by the weightedSum method using various weight values for the vectors generated with inlinks and outlinks. The *x*-axis represents the number of “the most similar” papers selected by the method and the *y*-axis the accuracy. Annotations such as “0.0 : 1.0” represent the weight balances between inlinks and outlinks. According to Figure 6, the accuracy of the weightedSum method tends to become higher when the weight for inlinks gets higher than the out-link weight. This is because the papers in the answer set are relatively famous ones frequently referenced by other papers. For this reason, we conducted the experiments using the “0.9 : 0.1” weight balance in order to obtain the best result.

Tables 3, 4, 5, 6, and 7 present the lists of top 10 papers found to be the most similar to a target paper, which is [33], using the three existing methods and the two proposed methods, respectively. (Note: the paper of [33] is concerned about clustering in data mining.) In the tables, those papers which are not similar to [33] are italic. In these papers, the authors mainly deal with issues of outlier detection or mining frequent patterns, indicating that the existing methods have made a wrong conclusion for these papers. In comparison, the results by the weightedSum method (shown in Table 6) include only one wrong entry (of frequent pattern mining) in the top 10 list, and the results by the undirected method do not include wrong papers (i.e., all related to clustering). These results imply that the undirected method performs better than the three existing methods and even than the weightedSum method. We have repeated this experiment many times with different target papers other than [33] and obtained results similar to Tables 3–7.

**Figure 7:**
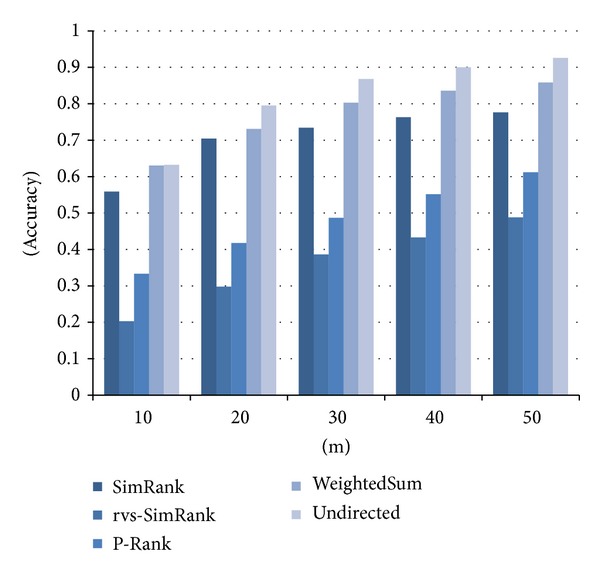
Accuracy of the similarity measures in scientific papers data.

Figure 7 compares the accuracy of similarities computed by the three existing methods and the two proposed methods. As in Figure 6, the *x*-axis represents the number of “the most similar” papers selected by each method and the *y*-axis the accuracy. The weightedSum method improved accuracy by 9% on average and up to 13% compared with SimRank. Also, the undirected method improved accuracy by 16% on average and up to 20% compared with SimRank. In conclusion, the two proposed methods turn out to compute the similarity more accurately than the existing methods. Especially, the undirected method performs the best.

### 5.3. Domain of Web Documents

**Figure 8:**
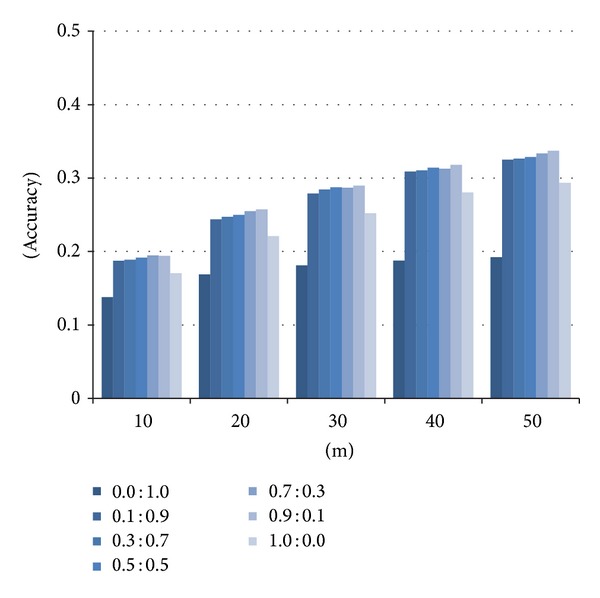
Accuracy of the weightedSum method with varying the weights.

Figure 8 shows the results of the accuracy on the similarities between Web pages obtained by the weightedSum method using various weight values between inlinks and outlinks. The *x*-axis represents the number of “the most similar” Web pages selected by the method and the *y*-axis the accuracy. Annotations such as “0.0 : 1.0” represent the weight balances between inlinks and outlinks. Again, the accuracy of the weightedSum method tends to become higher when the weight for inlinks gets higher, as in the case of scientific papers. This is because the answer set contains many Web pages with high authority. For this reason, we conducted the experiments using the “0.9 : 0.1” weight balance in order to obtain the best result.

**Figure 9:**
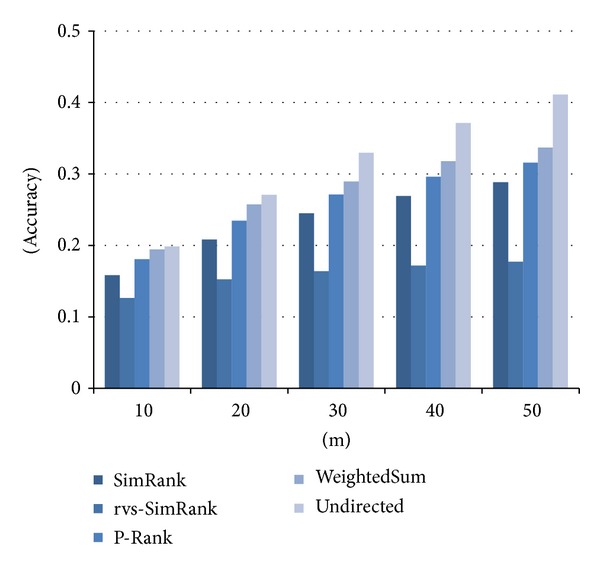
Accuracy of the similarity measures in Web documents data.

Figure 9 compares the accuracy of similarities computed by the three existing methods and the two proposed methods. Again, the *x*-axis represents the number of “the most similar” documents selected by each method, and the *y*-axis the accuracy. The weightedSum method improved accuracy by 20% on average and up to 24% compared with SimRank. Also, the undirected method improved accuracy by 34% on average and up to 43% compared with SimRank. In conclusion, the two proposed methods turn out to compute the similarity more accurately than the existing methods. Especially, the undirected method outperforms by a large degree the weightedSum and the three existing methods.

## 6. Conclusions

This paper presented new link-based similarity methods that can compute more accurately the similarity between objects by using the link information pertaining to the objects. Noticing that most existing link-based similarity methods use the pair-wise and level-wise models, we analyzed the problems with these models and proposed a new approach that does not suffer from these problems. In our proposed approach, each object is represented by a vector, all objects in the given universe as the elements of the vector, and a weight value to each object as the value of the corresponding element in the vector. As for this weight value, we proposed to utilize the notion of reachability between objects, computed using the “Random Walk with Restart” strategy. Then, we defined the similarity between two objects as the cosine similarity between two vectors representing the two objects. The proposed approach was then refined into two methods, the weightedSum and the undirected methods, differentiated by the strategy to handle the information on link directions. Examples showed that the two methods do not suffer from the problems of the pair-wise and level-wise models. In our experimentation of the proposed methods with the scientific papers and Web documents data sets, the results indicated that both of the proposed methods generally outperform the existing methods significantly.

## Figures and Tables

**Table 1:** Similarities between *a* and *b* and between *a*′ and *b*′ in Figure 2.

	rvs-SimRank	SimRank	P-Rank
*s*(*a*, *b*)	0.396	0	0.423
*s*(*a*′, *b*′)	0.321	0	0.408

**Table 2:** Similarities between *a* and *b* and between *a*′ and *b*′ using the proposed methods and existing methods in Figure 2.

	rvs-SimRank	SimRank	P-Rank	WeightedSum	Undirected
*s*(*a*, *b*)	0.396	0	0.423	1	1
*s*(*a*′, *b*′)	0.321	0	0.408	1	1

**Table 3:** Top 10 papers similar to [33] using SimRank.

First author	Title	Conference/journal	Year
Guha	CURE: An Efficient Clustering Algorithm for Large Databases	ACM SIGMOD	1998

Sheikholeslami	WaveCluster: A multi-Resolution Clustering Approach for Very Large Spatial Databases	VLDB	1998

Ester	*Knowledge Discovery in Large Spatial Databases: Focusing Techniques for Efficient Class Identification *	SSD	1995

Hinneburg	An Efficient Approach to Clustering in Large Multimedia Databases with Noise	AAAI	1998

Ng	Efficient and Effective Clustering Methods for Spatial Data Mining	VLDB	1994

Bradley	Scaling Clustering Algorithms to Large Databases	AAAI	1998

Wang	STING: A Statistical Information Grid Approach to Spatial Data Mining	VLDB	1997

L O'Callaghan	Streaming-Data Algorithms for High-Quality Clustering	IEEE ICDE	2002

Sander	A Density-Based Algorithm for Discovering Clusters in Large Spatial Databases with Noise	DMKD	1998

Arning	*A Linear Method for Deviation Detection in Large Databases *	ACM KDD	1996

**Table 4:** Top 10 papers similar to [33] using rvs-SimRank.

First author	Title	Conference/journal	Year
Knorr	* A Unified Notion of Outliers: Properties and Computation *	ACM KDD	1997

Guha	CURE: An Efficient Clustering Algorithm for Large Databases	ACM SIGMOD	1998

Sander	A Density-Based Algorithm for Discovering Clusters in Large Spatial Databases with Noise	DMKD	1998

Bradley	Scaling Clustering Algorithms to Large Databases	AAAI	1998

Guha	ROCK: A Robust Clustering Algorithm for Categorical Attributes	IEEE ICDE	1999

Sheikholeslami	WaveCluster: A multi-Resolution Clustering Approach for Very Large Spatial Databases	VLDB	1998

Arning	*A Linear Method for Deviation Detection in Large Databases *	ACM KDD	1996

Burdick	*MAFIA: A Maximal Frequent Itemset Algorithm* *for Transactional Databases *	IEEE TKDE	2005

Mannila	*Efficient Algorithms for Discovering Association Rules *	AAAI	1994

Kamber	*Metarule-Guided Mining of Multi-Dimensional* *Association Rules Using Data Cubes *	ACM KDD	1997

**Table 5:** Top 10 papers similar to [33] using P-Rank.

First author	Title	Conference/journal	Year
Knorr	* A Unified Notion of Outliers: Properties and Computation *	ACM KDD	1997

Guha	CURE: An Efficient Clustering Algorithm for Large Databases	ACM SIGMOD	1998

Sander	A Density-Based Algorithm for Discovering Clusters in Large Spatial Databases with Noise	DMKD	1998

Bradley	Scaling Clustering Algorithms to Large Databases	AAAI	1998

Sheikholeslami	WaveCluster: A multi-Resolution Clustering Approach for Very Large Spatial Databases	VLDB	1998

Guha	ROCK: A Robust Clustering Algorithm for Categorical Attributes	IEEE ICDE	1999

Mannila	*Efficient Algorithms for Discovering Association Rules *	AAAI	1994

Arning	* A Linear Method for Deviation Detection in Large Databases *	ACM KDD	1996

Silberschatz	*What Makes Patterns Interesting in Knowledge Discovery Systems *	IEEE TKDE	1996

Agrawal	*Mining Association Rules between Sets of Items in Large Databases *	ACM SIGMOD	1993

**Table 6:** Top 10 papers similar to [33] using weightedSum method.

First author	Title	Conference/journal	Year
Ester	*Knowledge Discovery in Large Spatial Databases:* *Focusing Techniques for Efficient Class Identification *	SSD	1995

Ng	Efficient and Effective Clustering Methods for Spatial Data Mining	VLDB	1994

Sheikholeslami	WaveCluster: A multi-Resolution Clustering Approach for Very Large Spatial Databases	VLDB	1998

Guha	ROCK: A Robust Clustering Algorithm for Categorical Attributes	IEEE ICDE	1999

Guha	CURE: An Efficient Clustering Algorithm for Large Databases	ACM SIGMOD	1998

Wang	STING: A Statistical Information Grid Approach to Spatial Data Mining	VLDB	1997

Hinneburg	An Efficient Approach to Clustering in Large Multimedia Databases with Noise	AAAI	1998

Aggarwal	Fast Algorithms for Projected Clustering	ACM SIGMOD	1999

Agrawal	Automatic Subspace Clustering of High Dimensional Data for Data Mining Applications	ACM SIGMOD	1998

Sander	A Density-Based Algorithm for Discovering Clusters in Large Spatial Databases with Noise	DMKD	1998

**Table 7:** Top 10 papers similar to [33] using undirected method.

First author	Title	Conference/journal	Year
Guha	CURE: An Efficient Clustering Algorithm for Large Databases	ACM SIGMOD	1998

Ng	Efficient and Effective Clustering Methods for Spatial Data Mining	VLDB	1994

Sander	A Density-Based Algorithm for Discovering Clusters in Large Spatial Databases with Noise	DMKD	1998

Bradley	Scaling Clustering Algorithms to Large Databases	AAAI	1998

Sheikholeslami	WaveCluster: A multi-Resolution Clustering Approach for Very Large Spatial Databases	VLDB	1998

Hinneburg	An Efficient Approach to Clustering in Large Multimedia Databases with Noise	AAAI	1998

Wang	STING: A Statistical Information Grid Approach to Spatial Data Mining	VLDB	1997

Agrawal	Automatic Subspace Clustering of High Dimensional Data for Data Mining Applications	ACM SIGMOD	1998

Aggarwal	Fast Algorithms for Projected Clustering	ACM SIGMOD	1999

Ankerst	OPTICS: Ordering Points To Identify the Clustering Structure	ACM SIGMOD	1999
